# Effects of Physical Training on Heart Rate Variability in Patients with Metabolic Syndrome: A Systematic Review and Meta-Analysis

**DOI:** 10.3390/jcm14176129

**Published:** 2025-08-29

**Authors:** Johan E. Ortiz-Guzmán, Manuel Sánchez-Soler, Laura Prieto-Mondragón, Óscar J. Arias-Mutis, Alexandra Bizy, Conrado J. Calvo, Antonio Alberola, Manuel Zarzoso

**Affiliations:** 1 Faculty of Health Sciences, University of Applied and Environmental Sciences (U.D.C.A), Bogotá 111166, Colombia; johortiz@udca.edu.co (J.E.O.-G.); laprieto@udca.edu.co (L.P.-M.); 2 Department of Physiology, University of Valencia, 46010 Valencia, Spain; masanso8@alumni.uv.es (M.S.-S.); conrado.calvo@uv.es (C.J.C.); antonio.alberola@uv.es (A.A.); 3 Department of Biomedical Sciences, CEU Cardenal Herrera, 46115 Valencia, Spain; oscar.j.arias@uv.es (Ó.J.A.-M.); alexandra.bizy@uchceu.es (A.B.); 4 Centro de Investigación Biomédica en Red de Enfermedades Cardiovasculares (CIBER-CV), 28029 Madrid, Spain; 5 CSIC-UPV, Instrumentation for Molecular Imaging Technologies Research Institute (I3M), Universitat Politècnica de València, 46022 Valencia, Spain; 6 Department of Physiotherapy, University of Valencia, 46010 Valencia, Spain

**Keywords:** heart rate variability, metabolic syndrome, physical training, endurance training, resistance training, HIIT, concurrent training

## Abstract

**Background/Objectives:** Heart rate variability (HRV) is a reliable, non-invasive marker of autonomic nervous system function and is often impaired in individuals with metabolic syndrome (MetS). Physical exercise has emerged as an effective strategy to improve autonomic modulation; however, the comparative effects of different training modalities on HRV in individuals with MetS remain unclear. This systematic review and meta-analysis aimed to evaluate the impact of various exercise interventions on HRV and to identify which training types yield the most significant improvements. **Methods**: A systematic search was conducted in PubMed and Scopus up to April 2025. Eligible studies (*n* = 16) included adults with obesity and MetS (*n* = 752) who underwent structured exercise interventions with HRV assessments pre- and post-intervention. Standardized mean differences were calculated using random effects models. Subgroup analyses were performed based on training modality (endurance training [ET], resistance training [RT], high-intensity interval training [HIIT], and concurrent training [CT]). **Results**: Sixteen studies of moderate to high quality were included, with eleven studies eligible for meta-analysis. ET and HIIT significantly improved time-domain indices (Root mean square of differences of successive R-R intervals —rMSSD—, Standard deviation of the R-R interval series —SDNN—) and frequency-domain parameters (high-frequency —HF—), suggesting enhanced parasympathetic activity. RT showed inconsistent effects, while CT improved long-term HF and total power (TP). Non-linear indices were the least reported due to insufficient data. **Conclusions**: Physical exercise—particularly ET and HIIT—appears to enhance cardiac autonomic modulation in individuals with obesity and MetS. These findings support incorporating targeted training strategies into clinical practice to optimize cardiovascular health in these populations.

## 1. Introduction

Metabolic syndrome (MetS) is a clinical condition characterized by the simultaneous presence of central obesity, arterial hypertension, atherogenic dyslipidemia, and insulin resistance [1]. This clustering of pathological conditions is associated with a significantly increased risk of cardiovascular disease [2], type 2 diabetes mellitus (T2DM) [3,4], and premature mortality [5]. Although the underlying pathophysiological mechanisms of these metabolic conditions are not yet fully elucidated, alterations in the normal functioning of the autonomic nervous system (ANS) appear as a key risk factor, reflected in an imbalance of autonomic function where sympathetic nervous system (SNS) activity predominates over parasympathetic nervous system (PNS) activity [6,7]. This imbalance has been associated with a progressive deterioration in cardiovascular control, increasing the risk of adverse cardiovascular events [8,9].

Given the extensive evidence regarding the relationship between autonomic dysfunction and metabolic conditions such as obesity and MetS, monitoring ANS activity through heart rate variability (HRV) has been widely recognized for its predictive value [10,11]. This non-invasive tool, based on the analysis of fluctuations between heartbeats, dynamically reflects the interaction between the ANS and cardiovascular function [12,13], allowing for the observation and quantification of autonomic cardiac balance. Therefore, HRV evaluation is useful both for diagnosis and for monitoring patients with MetS and its related pathologies. Several studies have employed long-term (24 h), short-term (5–15 min), and even ultra-short-term (10–20 s) recordings to assess the degree of autonomic dysfunction in this population, finding significant associations with arrhythmic events and risk of sudden cardiac death [11,14,15].

Physical exercise has been shown to be a fundamental non-pharmacological therapeutic intervention for managing various diseases, including those of metabolic origin, due to its ability to improve multiple cardiometabolic parameters [16]. In this context, moderate-to-high intensity endurance training (ET) has shown benefits in insulin sensitivity, blood pressure reduction, and lipid profile improvement [17,18]. Resistance training (RT) has also shown positive effects in this population by increasing muscle mass and improving body composition, which contributes to higher resting energy expenditure and enhanced glucose metabolism [19,20]. Moreover, high-intensity interval training (HIIT) has gained attention for its time efficiency and its positive effects on lipid oxidation and cardiorespiratory fitness [21], similar to the results observed with concurrent training (CT), which combines resistance and endurance modalities [22]. Collectively, this evidence supports the implementation of exercise programs tailored to individual characteristics as an effective strategy for managing pathological conditions like obesity and MetS. However, the effects of different training modalities on ANS modulation have not been systematically reviewed, nor have the differences in their impact on cardiovascular autonomic control been clearly defined and quantified. Furthermore, existing reviews have not compared training modalities or HRV domains specifically in MetS

Therefore, this systematic review and meta-analysis has two main objectives: first, to quantitatively evaluate the changes in HRV induced by physical training in patients with obesity and MetS, and second, to investigate whether different training modalities (ET, RT, HIIT, CT) exert distinct effects on HRV normalization following physical training.

## 2. Materials and Methods

### 2.1. Search Strategies

This study was prospectively registered in the International Prospective Register of Systematic Reviews (PROSPERO; registration ID: CRD420251018429) [link: https://www.crd.york.ac.uk/PROSPERO/view/CRD420251018429 (accessed on 4 July 2025)]. Its design and reporting followed the Preferred Reporting Items for Systematic Reviews and Meta-Analyses (PRISMA) guidelines [23]. Literature searches were performed in two major scientific databases—PubMed, hosted by the US National Library of Medicine, and Scopus—using keywords paired with corresponding MeSH terms relevant to the review question. The main search terms included “metabolic syndrome”, “obesity”, “heart rate variability”, and “HRV”, combined with “HIIT”, “endurance training”, and “resistance training”. Boolean operators (AND, OR) were applied to develop the search strings, restricting the search to the title and abstract fields (Table 1). Based on these search equations, we conducted the literature search between 15 January 2025 and 15 April 2025. No filters were applied regarding the year of publication.

### 2.2. Study Inclusion/Exclusion Criteria

The eligibility criteria for inclusion in this systematic review and meta-analysis were as follows: (i) original research articles; (ii) studies conducted in human participants; (iii) HRV measurements with recording durations ranging from 5 min to 24 h; (iv) availability of comparative data between pre- and post-intervention; (v) analysis of time-domain, frequency-domain, or non-linear HRV parameters; (vi) intervention periods exceeding two weeks; (vii) implementation of endurance training (ET), high-intensity interval training (HIIT), resistance training (RT), or combined training (CT); and (viii) publications written in English. A comprehensive description of the variables assessed is provided in Supplementary Table S1.

Studies were excluded if they were (i) systematic reviews and/or meta-analyses, (ii) narrative or bibliographic reviews, (iii) letters to the editor, or (iv) conference abstracts or communications.

### 2.3. Quality Assessment

The methodological quality of the included studies was assessed using the modified Standards for Reporting Diagnostic Accuracy Studies for HRV research (STARD-HRV) [24]. Unlike generic quality assessment tools, STARD-HRV incorporates criteria tailored to HRV research—such as detailed reporting of recording conditions, data processing procedures, and variability indices—which are critical for reproducibility and interpretation. Using this guideline ensures a more precise appraisal of methodological rigor and enhances comparability across HRV-related studies. This tool comprises 25 items, each valued at one point, for a maximum possible score of 25, and was adapted from the original STARD guidelines [25,26]. Based on the total score, studies were categorized as follows: ≤50% of the maximum score, “low methodological quality”; 51–75%, “good methodological quality”; and >75%, “excellent methodological quality. This approach ensured a standardized and objective assessment of study quality, thereby enhancing the reliability and transparency of the review process.

### 2.4. Data Extraction

The primary data from each study were extracted into a Microsoft Excel^®^ (2019) spreadsheet, including (i) general study information (title, authors, journal, year of publication, and study objective); (ii) participant characteristics (sample size by group, sex, age, body weight, height, body mass index [BMI], cardiorespiratory function, blood pressure, cholesterol levels, participation in weight loss programs, and maximal strength); (iii) HRV recording procedures (duration and timing of recordings, body position, ventilation control, fasting status, and assessment of time-domain, frequency-domain, and non-linear variables); (iv) diagnostic criteria applied for metabolic syndrome; and (v) the main outcomes of interest.

A total of 16 studies met the eligibility criteria and were included in the qualitative synthesis (systematic review, SR). Subsequently, a quantitative synthesis was conducted. The degree of heterogeneity was evaluated using the I^2^ statistic, following the recommendations of the Cochrane Handbook [27]: values ranging 0–40% indicated non-important heterogeneity; 30–60%, moderate heterogeneity; 50–90%, substantial heterogeneity; and 75–100%, considerable heterogeneity [27]. Particular attention was given to high heterogeneity in order to identify potential methodological discrepancies among the included studies. Furthermore, to minimize bias and reduce heterogeneity, studies reporting HRV recordings of less than 5 min or fewer than 250 intervals were excluded from the quantitative synthesis (MA) [9].

Then, to assess differences between groups, a meta-analysis (MA) was performed using Review Manager software version 5.4 for Windows (RevMan Version 5.4, The Nordic Cochrane Center, The Cochrane Collaboration, Copenhagen, Denmark) when at least two studies evaluated the same outcome. Prior to pooling, comparisons were organized as pre-intervention versus post-intervention. Differences between groups were quantified using Cohen’s d with corresponding 95% confidence intervals (CI) as the measure of standardized mean difference (SMD) when combining different outcome measures, and as mean difference (MD) when outcomes were assessed with the same measurement scale. Statistical significance values were also reported. When potential heterogeneity was detected (I^2^ > 50%), a random effects model was applied to calculate the pooled estimates. In contrast, when no significant heterogeneity was found, a fixed effects model was used to obtain the combined point estimates [28]. The effect size for each outcome was reported in the corresponding forest plot as SMD or MD. To facilitate interpretation, the magnitude of SMDs was classified according to Cohen’s conventions, considering values of 0.2 as small, 0.5 as moderate, and 0.8 as large. Given that each meta-analysis in this review included fewer than 10 studies, neither funnel plots nor Egger’s test were performed to assess publication bias, as recommended by the Cochrane Handbook [27]. Finally, subgroup analyses were conducted according to the “exercise type” factor. This analysis was only performed when at least two different types of training (i.e., ET, RT, HIIT, or CT) were reported separately across the included studies. HRV parameters with insufficient data were not included in the meta-analysis. We did not contact the authors for additional data.

## 3. Results

### 3.1. Identification of Studies

**Figure 1:**
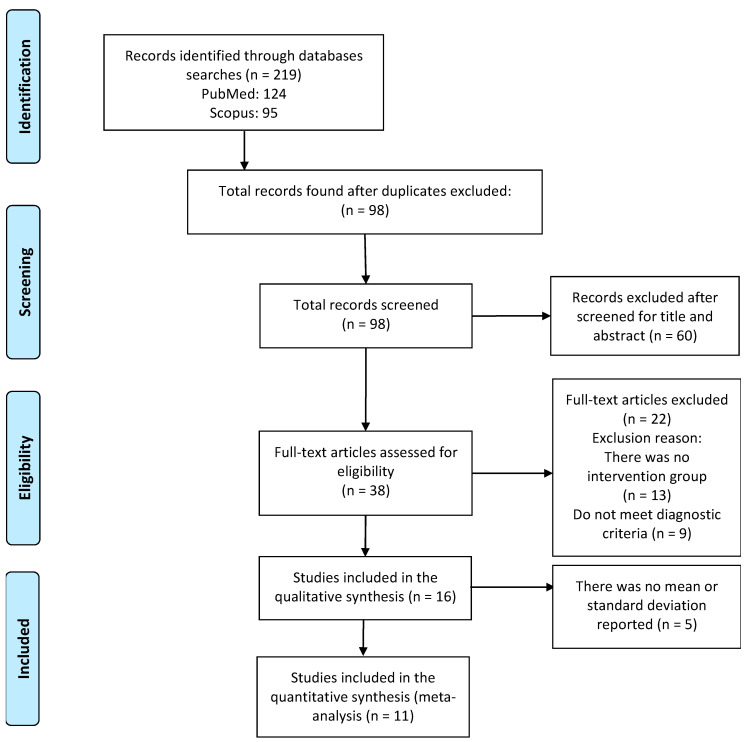
Flowchart of article selection.

The initial review identified 219 articles (124 in PubMed and 95 in Scopus). Duplicates across databases were removed, leaving a total of 98 articles. Titles and abstracts were screened, and 60 additional articles were excluded (49 for not measuring HRV, 10 for not being original research articles, and 1 for using animal models). Subsequently, 38 full-text articles were assessed for eligibility, and 16 met the inclusion criteria. No study was classified as having low methodological quality; therefore, all 16 were included in the qualitative synthesis. For the quantitative analysis (meta-analysis—MA), five studies were excluded due to missing mean and/or standard deviation data. Thus, the MA was conducted with 11 articles (Figure 1).

### 3.2. Methodological Quality Assessment

As a result of the methodological assessment, 12 articles (75%) were rated as “excellent methodological quality,” 4 articles (25%) as “good methodological quality,” and none were considered of “low methodological quality.” The average quality score across all studies was 76%, indicating an “excellent” overall methodological quality for the included articles. A detailed chart summarizing the methodological quality assessment for all included studies is available in the Supplementary Material (Table S2).

### 3.3. Study Characteristics

From the selected articles, the main characteristics of the participants and HRV recordings were extracted and are summarized in Table 2. Regarding publication year, two studies were published between 2000 and 2010, thirteen between 2013 and 2020, and one in 2024. Study populations came from the following regions: (a) North America (*n* = 2): Canada [29] and the United States [30]; (b) South America (*n* = 4): all from Brazil [31,32,33,34]; (c) Europe (*n* = 4): one each from Italy [35], Germany [36], Georgia [37], and France [38]; (d) Asia (*n* = 5): two from China [39,40], and one each from Nepal [41], Korea [42], and Thailand [43]; (e) Oceania (*n* = 1): from Australia [44].

In terms of age, 4 studies included participants aged 9 to 22 years [33,37,39,43], while 12 studies included adults aged 30 to 65 years [29,30,31,32,34,35,36,38,40,41,42,44]. Regarding sex, 2 studies included only women [30,42], 4 included only men [29,34,39,43], and the remaining 10 included both sexes [31,32,33,35,36,37,38,40,41,44]. A detailed table summarizing the mean age and sex distribution of the entire sample included in the review is available in the Supplementary Material (Table S3).

Regarding diagnostic criteria, six studies focused on MetS: two used the NCEP-ATP III criteria (National Cholesterol Education Program’s Adult Treatment Panel III) [29,34], three followed the IDF (International Diabetes Federation) guidelines [31,32,38], and one used the DFC (Diabetes Federation Criteria) [44]. Nine studies focused on individuals with obesity, with diagnosis based on BMI in five cases [35,36,40,42,43], triceps skinfold in one [37], the Expert Consensus on Obesity Prevention and Treatment in China in one [39], and unspecified criteria in two [30,33]. One study included individuals diagnosed with T2DM [41].

According to the criteria for quantitative analysis, the following variables were excluded from the MA (highlighted in black in Table 2): TINN, RRTri, SD2, SD1/SD2, α1, and α2. The remaining linear and nonlinear variables were included in the quantitative analysis (R-R, SDNN, rMSSD, pNN50, LF, HF, LF/HF, VLF, TP, SD1).

### 3.4. Characteristics of the Interventions

According to the inclusion criteria for this review, studies using endurance training (ET), resistance training (RT), high-intensity interval training (HIIT), and concurrent training (CT) protocols were selected. The main characteristics of these interventions are detailed in Table 3. Among the 16 studies analyzed, 2 used HIIT protocols [29,35], 6 applied ET [31,32,37,40,41,43], 3 used RT [30,33,34], and 3 included both HIIT and ET in separate groups [36,39,44]. The remaining two studies employed CT protocols [38,42].

The duration of the interventions varied: five studies lasted up to 8 weeks [29,35,38,39,41], four lasted 12 weeks [30,33,34,43], five lasted 16 weeks [31,32,36,40,44], one study lasted 24 weeks [42], and the remaining one lasted 32 weeks [37]. Training frequency ranged from three to five sessions per week, with duration ranging from 30 min in HIIT interventions to 120 min in CT protocols.

### 3.5. Effects of Training on Time-Domain HRV Variables

In total, 13 of the included studies analyzed time-domain HRV variables from short-term recordings. A summary of the main findings is presented in Table 4. Significant post-intervention increases in R-R intervals were found following HIIT [34] and ET [31,43]. With RT, one study reported significant increases [33], while another found no changes [34]. No significant effects were found in CT for any analyzed time-domain variables (R-R, SDNN, rMSSD) [42].

Additionally, 3 studies analyzed time-domain variables from long-term recordings (Table 5). HIIT produced significant increases in R-R intervals [35], as did CT [38]. However, the study by Stuckey et al. reported no significant changes in R-R intervals after 8 weeks of HIIT in participants with MetS [29].

**Figure 2:**
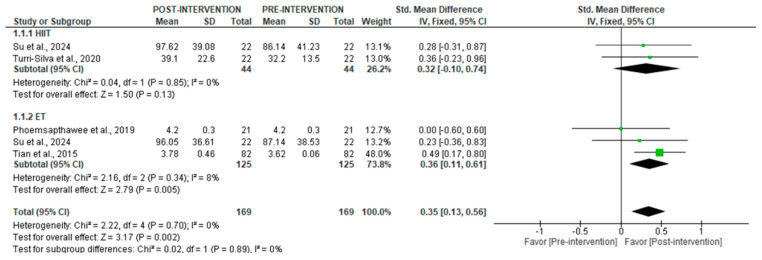
Forest plot of the effect of intervention on SDNN (pre- vs. post-intervention) in short-term recordings [34,39,40,43].

The results of the quantitative analysis showed that physical training significantly increased SDNN from short-term recordings post-intervention (SMD = 0.35 [95% CI = 0.13, 0.56], *p* = 0.002), with no heterogeneity across studies (I^2^ = 0%) (Figure 2). Subgroup analyses by training type showed significant increases for ET (SMD = 0.36 [95% CI = 0.11, 0.61], *p* = 0.005), but not for HIIT (*p* = 0.13).

**Figure 3:**
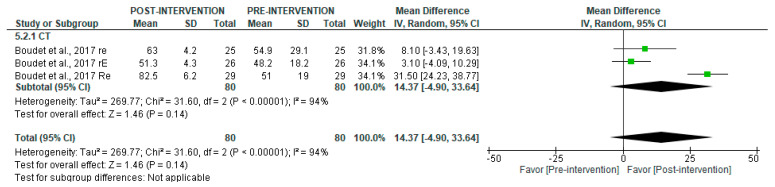
Forest plot of the effect of intervention on SDNN (pre- vs. post-intervention) in long-term recordings [38].

For long-term recordings (Figure 3), the MA could only be performed using CT data, which showed no significant changes between time points (*p* = 0.14), with high heterogeneity (I^2^ = 94%).

**Figure 4:**
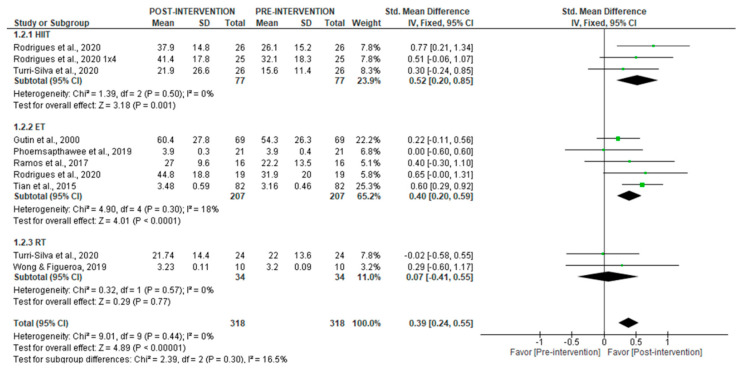
Forest plot of the effect of intervention on rMSSD (pre- vs. post-intervention) in short-term recording [30,34,36,37,40,43,44].

For rMSSD, Figure 4 (short-term recordings) shows a significant increase after the intervention period (SMD = 0.39 [95% CI = 0.24, 0.55], *p* < 0.00001), with no heterogeneity (I^2^ = 0%). Subgroup analyses revealed significant increases with both HIIT (SMD = 0.52 [95% CI = 0.20, 0.85], *p* = 0.001) and ET (SMD = 0.40 [95% CI = 0.20, 0.59], *p* < 0.00001), with low heterogeneity (I^2^ = 0% and 18%, respectively). No significant effects were found for RT (*p* = 0.77).

**Figure 5:**
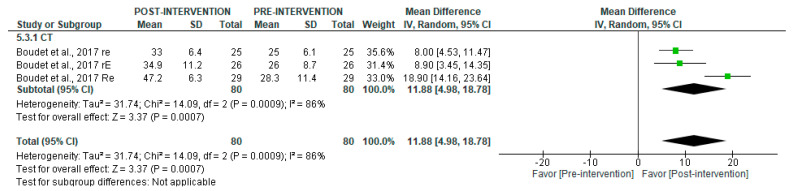
Forest plot of the effect of intervention on rMSSD (pre- vs. post-intervention) in long-term recording [38].

In long-term recordings (Figure 5), CT significantly increased rMSSD (MD = 11.88 [95% CI = 4.98, 18.78], *p* = 0.0007), despite high heterogeneity (I^2^ = 86%). Subgroup analyses were not possible for this variable.

**Figure 6:**
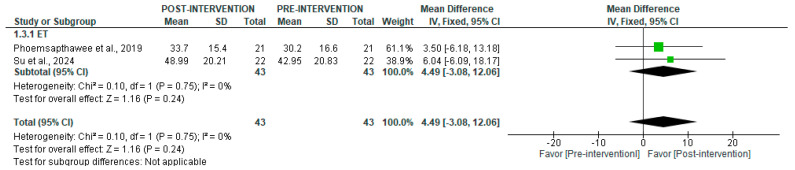
Forest plot of the effect of intervention on pNN50 (pre- vs. post-intervention) in short-term recording [39,43].

For pNN50, no significant changes were observed after ET in short-term recordings (*p* = 0.24) (Figure 6). Subgroup analyses could not be performed due to insufficient data from other training types.

**Figure 7:**
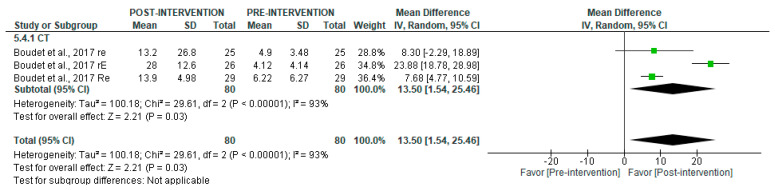
Forest plot of the effect of intervention on pNN50 (pre- vs. post-intervention) in long-term recording [38].

In contrast, the analysis of long-term recordings (Figure 7) showed a significant post-intervention increase with CT (MD = 13.50 [95% CI = 1.54, 25.46], *p* = 0.03), with high heterogeneity (I^2^ = 93%).

### 3.6. Effects of Training on Frequency-Domain HRV Variables

Of the 13 studies that analyzed short-term HRV recordings, 11 reported frequency-domain variables (Table 6), and 3 others used long-term recordings (Table 7).

**Figure 8:**
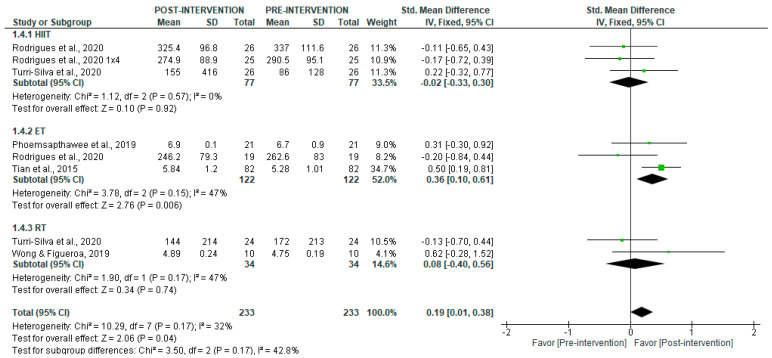
Forest plot of the effect of intervention on HF (pre- vs. post-intervention) in short-term recording [30,34,36,40,43].

Results from short-term data analysis showed a significant post-intervention increase in HF (SMD = 0.19 [95% CI = 0.01, 0.38], *p* = 0.04), with low heterogeneity (I^2^ = 32%) (Figure 8). Subgroup analyses revealed no significant changes in HF after HIIT (*p* = 0.92) or RT (*p* = 0.74), but a significant increase was observed with ET (SMD = 0.36 [95% CI = 0.10, 0.61], *p* = 0.006) (I^2^ = 47%).

**Figure 9:**
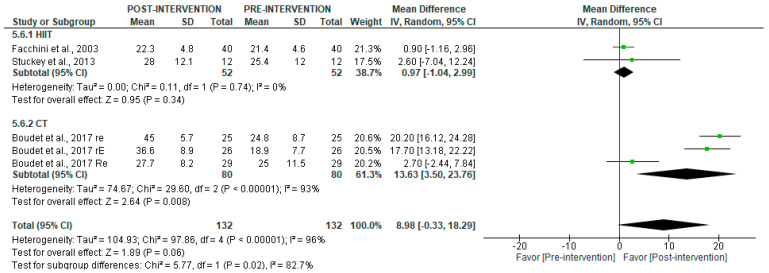
Forest plot of the effect of intervention on HF (pre- vs. post-intervention) in long-term recording [29,35,38].

Long-term recordings (Figure 9) showed no significant overall changes in HF post-intervention (*p* = 0.06), but the subgroup analyses indicated a significant increase after CT (MD = 13.63 [95% CI = 3.50, 23.76], *p* = 0.008), with no significant effect after HIIT (*p* = 0.34).

**Figure 10:**
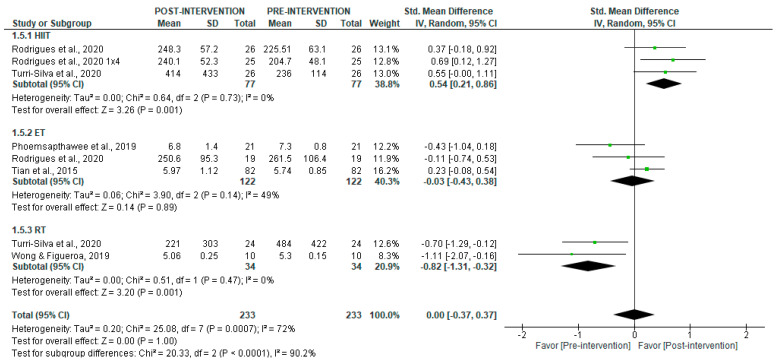
Forest plot of the effect of intervention on LF (pre- vs. post-intervention) in short-term recording [30,34,36,40,43].

Regarding LF in short-term recordings, no significant overall differences were observed pre- and post-intervention (*p* = 1.0), with high heterogeneity (I^2^ = 87%) (Figure 10). However, subgroup analyses revealed a significant post-intervention increase following HIIT (SMD = 0.54 [95% CI = 0.21, 0.86], *p* = 0.001), but no significant changes with ET (*p* = 0.89). In contrast, RT was associated with significantly lower LF values after the intervention (SMD = −0.82 [95% CI = −1.31, −0.32], *p* = 0.001).

**Figure 11:**
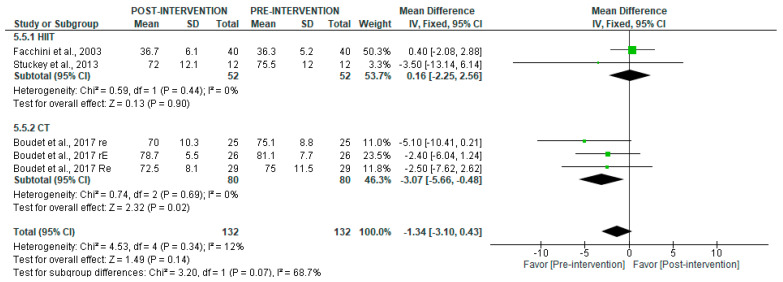
Forest plot of the effect of intervention on LF (pre- vs. post-intervention) in long-term recording [29,35,38].

The analysis of long-term recordings showed no overall significant changes (*p* = 0.14), but CT significantly decreased LF post-intervention (MD = −3.07 [95% CI = −5.66, −0.48], *p* = 0.02), while HIIT showed no significant difference (*p* = 0.90) (Figure 11).

**Figure 12:**
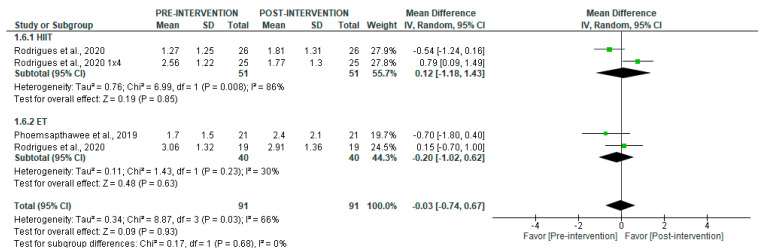
Forest plot of the effect of intervention on LF/HF (pre- vs. post-intervention) in short-term recording [36,43].

No significant changes were observed in the LF/HF ratio in short-term recordings after the intervention period (*p* = 0.93) (Figure 12), with moderate heterogeneity (I^2^ = 66%). Subgroup analyses revealed no significant differences for HIIT (*p* = 0.85) or ET (*p* = 0.63).

**Figure 13:**
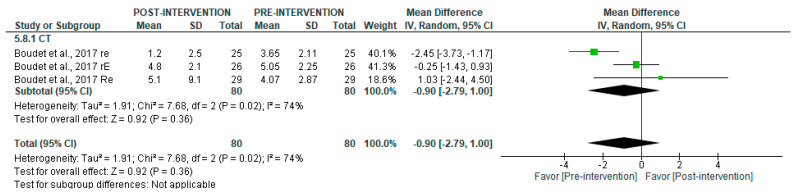
Forest plot of the effect of intervention on LF/HF (pre- vs. post-intervention) in long-term recording [38].

Similar results were found in long-term recordings, with no significant post-intervention changes (*p* = 0.36) and high heterogeneity (I^2^ = 74%) (Figure 13). Subgroup analysis was not feasible in this case.

**Figure 14:**
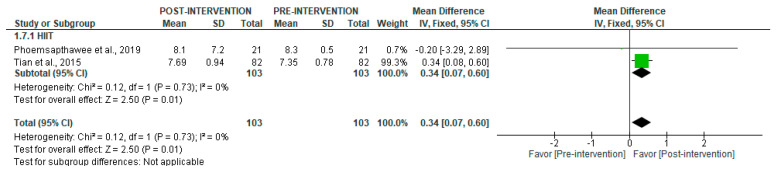
Forest plot of the effect of intervention on TP (pre- vs. post-intervention) in short-term recording [40,43].

Regarding total power (TP), short-term data showed a significant increase after HIIT (MD = 0.34 [95% CI = 0.07, 0.60], *p* = 0.01) (Figure 14).

**Figure 15:**
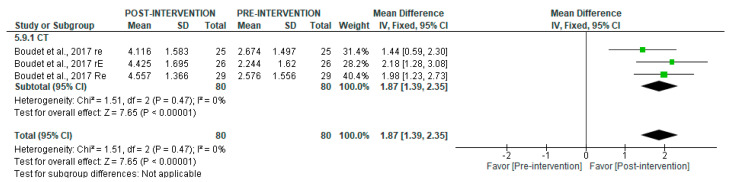
Forest plot of the effect of intervention on TP (pre- vs. post-intervention) in long-term recording. Values in s^2^ (divided ms^2^ by 1000 for graphical representation purposes) [38].

With respect to long-term recordings, CT was associated with a significant post-intervention increase (MD = 1868.07 [95% CI = 1389.32, 2346.81], *p* < 0.00001) (Figure 15). For this variable, it was not possible to perform subgroup analyses in either short- or long-term recordings.

**Figure 16:**
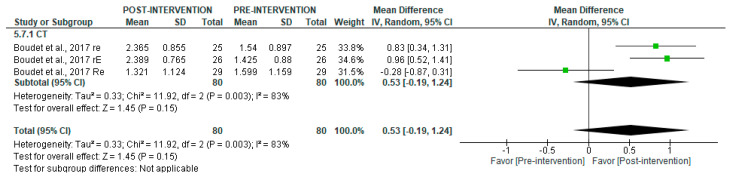
Forest plot of the effect of intervention on VLF (pre- vs. post-intervention) in long-term recording. Values in s^2^ (divided ms^2^ by 1000 for graphical representation purposes) [38].

For very low frequency (VLF), only long-term data were available. The meta-analysis showed no significant changes following CT (*p* = 0.15), with high heterogeneity (I^2^ = 83%) (Figure 16).

### 3.7. Effects of Training on Non-Linear HRV Variables

Among the 13 studies that analyzed short-term recordings, 6 reported non-linear HRV variables (Table 8). The main qualitative findings are summarized in Table 8. Regarding Poincare plot variables, two studies found significant increases in SD2 following HIIT [36,44], and three after ET [31,36,41]. However, one study on HIIT [34] and another on ET [44] reported no significant post-intervention changes. Conversely, Goit et al. and Turri et al. found a significant reduction in SD2 following ET [41] and RT [34], respectively. The SD1/SD2 ratio was not significantly modified after HIIT [44] or ET [31,43,44]. However, Goit et al. reported a significant reduction in this ratio after 6 weeks of ET [41]. Additionally, Ramos et al. [44] analyzed DFA variables but found no significant changes in α1 or α2 following the intervention program, regardless of whether HIIT or ET was applied. No studies reported non-linear HRV outcomes using long-term recordings.

**Figure 17:**
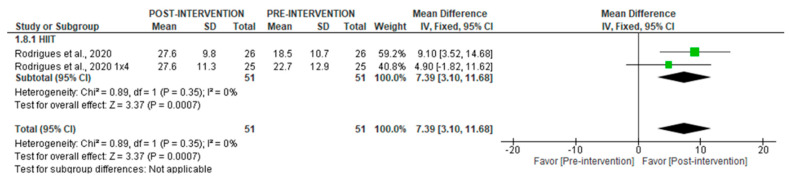
Forest plot of the effect of intervention on SD1 (pre- vs. post-intervention) in short-term recording [36].

The meta-analysis showed a significant post-intervention increase in SD1 following HIIT (MD = 7.39 [95% CI = 3.10, 11.68], *p* = 0.0007) (I^2^ = 0%) (Figure 17). No quantitative analysis could be performed for any other non-linear variables.

## 4. Discussion

We conducted this systematic review and meta-analysis to evaluate the effects of different exercise training modalities on HRV in individuals with obesity and MetS, as well as to determine whether these training modalities exert distinct impacts on autonomic modulation. A total of 16 moderate-to-high-quality studies were reviewed, 11 of which were included in the quantitative synthesis.

The main findings were as follows: (1) physical training significantly improved parasympathetic-related HRV indices such as rMSSD, SDNN, and HF; (2) RT showed less consistent effects, with limited improvement in time-domain indices and no significant changes in frequency-domain parameters; (3) CT promoted favorable changes, particularly in HF and total power in long-term recordings; (4) when subgroup analyses were possible, the results showed that ET and HIIT exert the greatest impact on HRV, particularly by enhancing parasympathetic activity; (5) non-linear HRV variables were the least studied across the included trials, primarily due to the limited availability of data (only six studies of short-term HRV were found), which prevented consistent quantitative analysis and generalization of findings. Nevertheless, SD1 improved with HIIT, showing the improvement in parasympathetic regulation.

The observed increases in rMSSD and SDNN following ET corroborate previous findings indicating enhanced vagal tone after aerobic exercise interventions [9,45]. These time-domain indices are widely recognized as indicators of parasympathetic activity and cardiovascular health [46]. The superior efficacy of ET over other modalities in increasing SDNN may be attributed to its sustained and rhythmic nature, which likely facilitates vagal reactivation post-exercise [47]. Notably, an improvement in SDNN has significant clinical implications. Low SDNN values are robust predictors of both cardiovascular morbidity and all-cause mortality, with studies showing approximately a 1% reduction in cardiovascular risk per 1 ms increase in SDNN [48,49]. The capacity of ET and HIIT to enhance this parameter highlights their potential not only to improve autonomic balance but also to reduce long-term cardiovascular risk in individuals with MetS and related conditions. Furthermore, increases in SDNN may reflect enhanced adaptability of the cardiac autonomic system to physiological stressors, suggesting a broader benefit of endurance-based interventions in terms of cardiovascular resilience and health outcomes. These findings reinforce the clinical relevance of prioritizing exercise prescriptions that effectively target vagal function, especially in populations at elevated cardiometabolic risk.

Our results also indicate that HIIT significantly increases rMSSD and SD1, supporting its role as a time-efficient alternative for improving vagal modulation [50]. HIIT has been shown to elicit rapid autonomic adaptations due to its repeated exposure to high-intensity effort and recovery phases [51]. Nonetheless, the limited effects observed for RT suggest that resistance-based interventions may have less impact on HRV parameters, possibly due to their transient activation of sympathetic responses during lifting and reduced vagal activation post-exercise [52,53]. The timing and magnitude of transient sympathetic activation post-exercise are critical factors in understanding how the ANS responds to exercise stress and regulates recovery. This sympathetic surge helps facilitate the restoration of metabolic function, vascular tone, and energy balance [54]. However, the duration and intensity of this activation can vary depending on factors like exercise intensity, fitness level, and training status, with trained individuals generally recovering more quickly due to enhanced parasympathetic activity [55,56].

Frequency-domain analysis revealed that both ET and CT protocols significantly increased HF power, an index closely linked to respiratory sinus arrhythmia and vagal tone [9]. These findings support the hypothesis that aerobic components are more effective in enhancing parasympathetic modulation at rest. The observed improvements in HF power likely reflect physiological adaptations such as enhanced baroreflex sensitivity, reductions in visceral adiposity and systemic inflammation, and increased cardiac vagal outflow [46]. In contrast, RT’s inconsistent outcomes may reflect variations in protocol design (e.g., load, rest intervals), participant training status, or insufficient volume to elicit meaningful changes in autonomic tone [53].

Although the LF/HF ratio is frequently interpreted as a marker of sympathovagal balance, its physiological meaning remains controversial, particularly in the context of exercise interventions. The present meta-analyses showed inconsistent or non-significant changes in LF/HF ratio across training modalities, despite observable changes in other HRV indices. This discrepancy highlights the need to interpret the LF/HF ratio with caution, especially considering its inherent methodological and physiological limitations. The LF/HF ratio is commonly used as a marker of sympathovagal balance, with the LF band often linked to sympathetic activity and HF to parasympathetic modulation. However, the ratio’s interpretation has been questioned due to the overlap in the contributions of both autonomic branches to LF and the influence of breathing patterns and baroreflex sensitivity. This makes the LF/HF ratio context-dependent, particularly as LF can also reflect parasympathetic modulation under certain conditions, such as controlled breathing [9,46]. Additionally, the complexity of LF power, its weak correlation with sympathetic nerve activation, and the non-linear (and often non-reciprocal) interactions between sympathetic and parasympathetic activity, which are influenced by factors like respiratory mechanics and heart rate, make it difficult to accurately determine the physiological basis of the LF/HF ratio [57]. Furthermore, the decrease in LF power in HRV is context-dependent. In healthy individuals, a decrease might reflect improved parasympathetic dominance, which is generally considered beneficial [46]. However, in individuals with certain pathologies (e.g., heart failure, chronic fatigue, or autonomic dysfunction), a decrease in LF power might reflect sympathetic underactivity, which can be detrimental and associated with poor health outcomes [9].

The differential effects observed across training modalities in HRV outcomes may be attributed to distinct underlying physiological mechanisms. HIIT and ET protocols consistently enhanced parasympathetic modulation and decreased sympathetic drive, as reflected by significant increases in rMSSD, SD1, and HF power in several included studies. These adaptations are likely mediated by improvements in baroreflex sensitivity, reductions in systemic inflammation and visceral adiposity, and increased cardiac vagal activity [39,41,44]. In contrast, resistance training protocols showed more limited or heterogeneous effects, often restricted to time-domain indices such as rMSSD and SDNN, possibly due to transient sympathetic activation during muscle contraction and a blunted parasympathetic rebound post-exercise [30,33]. Studies using combined or periodized approaches tended to report broader improvements, likely due to the integration of sustained aerobic stimuli and neuromuscular load, which may enhance autonomic plasticity via both central and peripheral adaptations [42,43]. Overall, these findings suggest that the autonomic benefits of exercise are modality- and intensity-dependent and highlight the relevance of vagal-driven HRV indices as sensitive markers of training responsiveness.

Importantly, improvements in HRV may translate to clinically relevant outcomes, as higher HRV has been linked to lower cardiovascular mortality and improved metabolic profiles [58]. Therefore, promoting exercise modalities that enhance HRV could play a crucial role in managing patients with metabolic dysfunction.

From a methodological perspective, the high heterogeneity observed in several analyses is a limitation, likely stemming from differences in HRV measurement methods, sample characteristics, inclusion of both sexes in the same analysis and intervention duration. According to the GRADE framework [59], this substantial inconsistency reduces the certainty of the overall effect estimates, and therefore, the results should be interpreted with caution. Although subgroup analyses were performed for several variables, insufficient data in others limited further exploration. This is consistent with findings from previous systematic reviews in MetS populations, where sex-specific autonomic responses were identified [6,7]. Standardization of HRV protocols, including recording length and posture, is essential for comparability across studies [9,60].

In conclusion, this study reinforces the role of physical training—particularly ET and HIIT—as effective strategies for improving autonomic function in populations with MetS, obesity, and T2DM. In clinical settings, individualized exercise prescriptions prioritizing ET and HIIT could enhance autonomic health and reduce cardiometabolic risk, especially if stratified by sex. Further longitudinal trials with standardized HRV protocols, accounting for recording duration, body position, and breathing rate, are needed to better understand the long-term implications of these adaptations.

## Figures and Tables

**Table 1:** Search equation.

Database	Search Equation
PubMed	((“heart rate variability” [Title/Abstract] OR “HRV” [Title/Abstract]) AND (“endurance training” [Title/Abstract] OR “HIIT” [Title/Abstract] OR “resistance training” [Title/Abstract]) AND (“metabolic syndrome” [Title/Abstract] OR “obesity” [Title/Abstract]))
Scopus	((TITLE-ABS-KEY (“metabolic syndrome”) OR TITLE-ABS-KEY (“obesity”)) AND (TITLE-ABS-KEY (“heart rate variability”) OR TITLE-ABS-KEY (“HRV”)) AND (TITLE-ABS-KEY (“endurance training”) OR TITLE-ABS-KEY (“HIIT”) OR TITLE-ABS-KEY (“resistance training”))

**Table 2:** Summary of the studies’ characteristics included in the review.

Reference	Methodological Evaluation (%)	*n*	Age (Years)	Gender	Diagnostic Criteria	Recording Characteristics		Analyzed HRV Variables		
						Recording Time	Body Position	Time	Frequency	Non-Linear
(Stuckey et al., 2013) [29] ‡	68%	12	50–64	Men	ATP III	24 h	NR	R–R	LF, HF, LF/HF	No
(Wong & Figueroa, 2019) [30] ‡	84%	20	50–65	Women	NR	5 min	Supine	R–R, rMSSD	TP, LF, HF, LF/HF	No
(Vanzella, Dagostinho et al., 2019) [31]	68%	52	44–56	Both	IDF	30 min	Supine	R–R, **TINN, rRTRI**	No	SD1, **SD2, SD1/SD2**
(Vanzella, Linares et al., 2019) [32]	88%	53	44–56	Both	IDF	30 min	Supine	R–R, rMSSD, SDNN	LF, HF, LF/HF	No
(Farinatti et al., 2016) [33]	72%	44	NR	Both	NR	5 min	NR	R–R, SDNN, rMSSD	LF, HF, LF/HF	No
(Turri-Silva et al., 2020) [34] ‡	88%	38	51–52	Men	NCEP-ATP III	20 min	Supine	R–R, SDNN, rMSSD, **TINN, RRTri**	LF, HF	SD1, **SD2**
(Facchini et al., 2003) [35] ‡	76%	40	23–37	Both	BMI > 41 Kg/m^2^	18 h	NR	R–R, SDNN, rMSSD, pNN50	VLF, LF, HF, LF/HF	No
(Rodrigues et al., 2020) [36] ‡	88%	70	31–58	Both	BMI > 24 Kg/m^2^	10 min	Supine	rMSSD	LF, HF, LF/HF	SD1, **SD2**
(Gutin et al., 2000) [37] ‡	72%	79	9–10	Both	Triceps skinfold *	10 min	Supine	rMSSD	No	No
(Boudet et al., 2017) [38] ‡	76%	80	50–70	Both	IDF	24 h	NR	R–R, SDNN, rMSSD, pNN50	LF, HF, VLF	No
(Su et al., 2024) [39] ‡	68%	44	13–15	Men	¤	5 min	Standing	R–R, SDNN, rMSSD, pNN50	LF, HF, LF/HF	No
(Tian et al., 2015) [40] ‡	84%	82	42	Both	BMI > 24 Kg/m^2^	10 min	Supine	R–R, rMSSD, SDNN	TP, HF, LF	No
(Goit et al., 2018) [41]	76%	41	40–49	Both	NR	5 min	Supine	R–R, SDNN, rMSSD, pNN50	HF, LF, LF/HF	SD1, **SD2, SD1/SD2**
(Kim et al., 2018) [42]	60%	20	65–66	Women	BMI > 30 kg/m^2^ and/or body fat percentage > 30%	10 min	NR	R–R, SDNN, rMSSD	TP, LF, HF, LF/HF	No
(Phoemsapthawee et al., 2019) [43] ‡	88%	21	19–22	Men	BMI > 25 kg/m^2^ and/or body fat percentage > 24%	5 min	Supine	R–R, SDNN, rMSSD, pNN50	LF, HF, LF/HF	SD1, **SD2, SD1/SD2**
(Ramos et al., 2017) [44] ‡	80%	56	>30	Both	DFC	5 min	Supine	R–R, SDNN, rMSSD, pNN50	LF, HF, LF/HF	SD1, **SD2, α1, α2**

NCEP-ATP III: National Cholesterol Education Program’s Adult Treatment Panel III. IDF: International Diabetes Federation. DFC: diabetes federation criteria *: triceps skinfold above the 85th percentile for age, sex, and ethnicity. ¤ Expert Consensus on Obesity Preventy and Treatment in China. NR: not reported. ‡: Included in the meta-analysis.

**Table 3:** Characteristics of the interventions.

Reference	Type of Training	Intervention Duration (Weeks)	Frequency (Days/Week)	Training Time (min)
(Stuckey et al., 2013) [29] ‡	HIIT	8	3	NR
(Wong & Figueroa, 2019) [30] ‡	RT	12	3	NR
(Vanzella, Dagostinho et al., 2019) [31]	ET	16	3	30–75
(Vanzella, Linares et al., 2019) [32]	ET	16	3	30–75
(Farinatti et al., 2016) [33]	RT	12	3	30–40
(Turri-Silva et al., 2020) [34] ‡	HIIT	12	3	40
	RT	12	3	40
(Facchini et al., 2003) [35] ‡	HIIT	3	5	35
(Rodrigues et al., 2020) [36] ‡	HIIT	16	3	40
	ET	16	3	30
(Gutin et al., 2000) [37] ‡	ET	32	NR	NR
(Boudet et al., 2017) [38] ‡	CT	3	3	15 + NR
(Su et al., 2024) [39] ‡	HIIT	8	3	30
	ET	8	3	30
(Tian et al., 2015) [40] ‡	ET	16	3	40–60
(Goit et al., 2018) [41]	ET	6	3	50
(Kim et al., 2018) [42]	CT	24	4	30 + 120
(Phoemsapthawee et al., 2019) [43] ‡	ET	12	4	60
(Ramos et al., 2017) [44] ‡	HIIT	16	3	38
	ET	16	6	30

HIIT: High Intensity Interval Training. ET: Endurance training. RT: Resistance training. CT: Concurrent training. NR: not reported. ‡: Included in the meta-analysis.

**Table 4:** Modifications in short-term HRV recordings (time domain) in Metabolic Syndrome, Obesity, and Type 2 Diabetes according to the type of training.

Reference	HIIT				ET				RT				CT		
	R-R	SDNN	rMSSD	pNN50	R-R	SDNN	rMSSD	pNN50	R-R	SDNN	rMSSD	pNN50	R-R	SDNN	rMSSD
(Wong & Figueroa, 2019) [30]											=				
(Vanzella, Dagostinho et al., 2019) [31]					↑										
(Vanzella, Linares et al., 2019) [32]						↑	↑								
(Farinatti et al., 2016) [33]									↑	↑	↑	↑			
(Turri-Silva et al., 2020) [34]	↑	↑	↑						=	↓	=				
(Rodrigues et al., 2020) [36]			↑				↑								
(Gutin et al., 2000) [37]							↑								
(Su et al., 2024) [39]		↑	↑	↑		↑	↑	↑							
(Tian et al., 2015) [40]						↑	↑								
(Goit et al., 2018) [41]						↑	↑	↑							
(Kim et al., 2018) [42]													=	=	=
(Phoemsapthawee et al., 2019) [43]					↑	=	=	↑							
(Ramos et al., 2017) [44]		↑	↑	↑		=	=	=							

↑: increases as a result of the intervention; ↓: decreases as a result of the intervention; =: no change as a result of the intervention.

**Table 5:** Modifications in long-term HRV recordings (time domain) in Metabolic Syndrome according to the type of training.

Reference	HIIT				CT			
	R-R	SDNN	rMSSD	pNN50	R-R	SDNN	rMSSD	pNN50
(Stuckey et al., 2013) [29]	=							
(Facchini et al., 2003) [35]	↑	↑	↑	↑				
(Boudet et al., 2017) [38]					↑	↑	↑	↑

↑: increases as a result of the intervention; =: no change as a result of the intervention.

**Table 6:** Modifications in short-term HRV recordings (frequency domain) in Metabolic Syndrome, Obesity, and Type 2 Diabetes according to the type of training.

Reference	HIIT				ET				RT				CT		
	LF	HF	LF/HF	TP	LF	HF	LF/HF	TP	LF	HF	LF/HF	TP	LF	HF	LF/HF
(Wong & Figueroa, 2019) [30]					↑				↑	↑	↓	=			
(Vanzella, Linares, et al., 2019) [32]					↑	=	=								
(Farinatti et al., 2016) [33]									↑	↑	↓	↑			
(Turri-Silva et al., 2020) [34]	↑	↑							↓	↓					
(Rodrigues et al., 2020) [36]	↑	↓	=		↓	↓	=								
(Su et al., 2024) [39]	↑	↑	↓		↑	↑	↓								
(Tian et al., 2015) [40]					↑	↑		↑							
(Goit et al., 2018) [41]					↑	↑	↓								
(Kim et al., 2018) [42]													↓	=	=
(Phoemsapthawee et al., 2019) [43]					=	↑	↓	=							
(Ramos et al., 2017) [44]	↑	↑	=		=	=	=								

↑: increases as a result of the intervention; ↓: decreases as a result of the intervention; =: no change as a result of the intervention.

**Table 7:** Modifications in long-term HRV recordings (frequency domain) in Metabolic Syndrome according to the type of training.

Reference	HIIT				CT			
	LF	HF	VLF	LF/HF	LF	HF	VLF	LF/HF
(Stuckey et al., 2013) [29]	↓	↑		↓				
(Facchini et al., 2003) [35]	↑	↑	↑	=				
(Boudet et al., 2017) [38]					↓	↑	↑	↓

↑: increases as a result of the intervention; ↓: decreases as a result of the intervention; =: no change as a result of the intervention.

**Table 8:** Modifications in short-term HRV recordings (non-linear analysis) in Metabolic Syndrome, Obesity, and Type 2 Diabetes according to the type of training.

Reference	HIIT					ET					RT	
	SD1	SD2	SD1/SD2	α1	α2	SD1	SD2	SD1/SD2	α1	α2	SD1	SD2
(Vanzella, Dagostinho, et al., 2019) [31]						↑	↑	=				
(Turri-Silva et al., 2020) [34]	↑	=									=	↓
(Rodrigues et al., 2020) [36]	↑	↑				↑	↑					
(Goit et al., 2018) [41]						↑	↑	↓				
(Phoemsapthawee et al., 2019) [43]						=	↓	=				
(Ramos et al., 2017) [44]	↑	↑	=	=	=	=	=	=	=	=		

↑: increases as a result of the intervention; ↓: decreases as a result of the intervention; =: no change as a result of the intervention.

## Data Availability

All data generated or analyzed during this study are included in the published studies and Supplementary Information.

## Supplementary Materials

The following supporting information can be downloaded at: https://www.mdpi.com/article/10.3390/jcm14176129/s1, Table S1: Description of main HRV measurements included in the study. Table S2: Methodological quality assessment for all included studies (STARD-HRV). Table S3: Summary of the samples’ characteristics included in the review.

## Abbreviations

The following abbreviations are used in this manuscript:

HRV	Heart rate variability
MetS	Metabolic syndrome
ET	Endurance training
RT	Resistance training
HIIT	High-intensity interval training
CT	Concurrent training
STARD	Standard for Reporting Diagnostic Accuracy Studies
LF	Low-frequency (0.04–0.15 Hz)
HF	High-frequency (0.15–0.4 Hz)
VLF	Very-low frequency (<0.04 Hz)
TP	Total power
SDNN	Standard deviation of the R-R interval series
RMSSD	Root mean square of differences of successive R-R intervals
