# Supplementary material for: Effects of Physical Training on Heart Rate Variability in Patients with Metabolic Syndrome: A Systematic Review and Meta-Analysis
Source: J Clin Med. 2025 Aug 29;14(17):6129. doi: 10.3390/jcm14176129 (PMC12429106; doi:10.3390/jcm14176129)
Supplement: Supplementary file 1 [file jcm-14-06129-s001.zip › jcm-3793917-supplementary.pdf]

Table S1. Description of main HRV measurements included in the study [1–3].

PARAMETER	DESCRIPTION
**Time-domain measurements**	
R-R- interval (ms)	Time period between successive heartbeats (interbeat interval).
SDNN (ms)	Standard deviation of the R-R interval series (overall variability). SDNN reflects all the cyclic components responsible for variability in the period of recording. Both sympathetic nervous system (SNS) and parasympathetic nervous system (PNS) activity might contribute to SDNN.
rMSSD (ms)	The root mean square of differences of successive R-R intervals. The RMSSD reflects the beat-to-beat variance in HR and may be the primary time-domain measure used to estimate the vagally mediated changes reflected in HRV.
pNN50 (percentage)	Number of adjacent NN intervals that differ from each other by more than 50 ms. This parameter seems to be closely correlated with PNS activity.
**Frequency-domain measurements**	
LF, HF	Ranges of the spectral components of the HRV:<br>• LF: low frequency (0.04 – 0.15 Hz). LF power may be produced by both the PNS and SNS, and blood pressure regulation via baroreceptors.<br>• HF: high frequency (0.15 – 0.4 Hz). The efferent vagal activity is a major contributor to the HF component and it is known as the respiratory band because it corresponds to the HR variations related to the respiratory cycle.
LF/HF	Ratio between LF and HF bands. Considered to mirror sympathovagal balance.
TP	In the frequency-domain analysis of Heart Rate Variability (HRV), Total Power (TP) refers to the overall variance of the HRV signal across a defined frequency range. It is usually expressed in milliseconds squared (ms^2) and represents the aggregate energy contained in the different components of the HRV spectrum
**Non-linear measurements**	
DFA-α1(a.u.)	Detrended fluctuation analysis. Describes short-term fluctuations, mainly mediated by the baroreceptor reflex.
DFA-α2 (a.u.)	Detrended fluctuation analysis. Describes long-term fluctuations and reflects the regulatory mechanisms that limit fluctuation of the beat cycle.
Poincaré Plot:<br>SD1 (ms)<br>SD2 (ms)<br>SD1/SD2 (a.u.)	SD1: Poincaré plot standard deviation perpendicular to the line of identity. Measures short-term HRV in ms and correlates with baroreflex sensitivity (BRS). The RMSSD is identical to the non-linear metric SD1, which reflects short-term HRV.<br>SD2: Poincaré plot standard deviation along the line of identity. Measures short- and long-term HRV in ms and correlates with LF power and BRS.<br>Ratio between SD1 and SD2, correlated with the LF/HF ratio and used to measure autonomic balance when the monitoring period is sufficiently long.

Table S2. Methodological quality assessment for all included studies (STARD-HRV) [20].

	1	2	3	4	5	6	7	8	9	10	11	12	13	14	15	16	17	18	19	20	21	22	23	24	25		
					METHODS															RESULTS							
REFERENCE	TITLE	ABSTRACT	INTRODUCTION		Study design		Participants	Pre-Testing	Test methods			Interbeat interval		Analysis						Participants	Test results	DISCUSSION		OTHER INFORMATION		TOTAL	
(Gutin et al., 2000) [4]	1	1	1	1	1	0	1	0	1	0	1	1	0	1	1	0	0	1	1	1	1	0	1	1	1	18	72%
(Facchini et al., 2003) [5]	1	1	1	1	1	0	0	0	1	1	0	1	1	1	1	1	1	1	0	1	1	0	1	1	1	19	76%
(Stuckey et al., 2013) [6]	1	1	1	1	1	0	1	0	1	1	0	1	0	0	1	0	0	1	1	1	1	1	1	0	1	17	68%
(Tian et al., 2015) [7]	1	1	1	1	1	0	1	1	1	1	1	1	1	0	1	1	1	0	0	1	1	1	1	1	1	21	84%
(Farinatti et al., 2016) [8]	1	1	1	1	1	0	1	0	1	0	0	1	0	1	0	0	1	1	1	1	1	1	1	1	1	18	72%
(Boudet et al., 2017) [9]	1	1	1	1	0	0	1	1	1	0	0	0	0	1	1	1	1	1	1	1	1	1	1	1	1	19	76%
(Ramos et al., 2017) [10]	1	1	1	1	1	1	1	1	0	1	1	1	1	0	1	0	1	0	0	1	1	1	1	1	1	20	80%
(Goit et al., 2018) [11]	1	1	1	1	1	0	1	1	1	0	1	0	0	1	1	1	0	0	1	1	1	1	1	1	1	19	76%
(KIM et al., 2018) [12]	1	1	1	1	1	1	1	1	0	1	1	0	1	0	0	0	0	0	0	1	1	0	1	0	1	15	60%
(Phoemsapthawee et al., 2019) [13]	1	1	1	1	1	0	1	1	1	1	1	1	1	1	0	1	1	1	1	1	1	1	1	0	1	22	88%
(Vanzella, Dagostinho, et al., 2019) [14]	1	1	1	1	1	0	1	0	1	1	1	0	1	1	0	0	0	0	1	1	1	1	1	1	0	17	68%
(Vanzella, Linares, et al., 2019) [15]	1	1	1	1	1	1	1	0	1	1	1	1	1	0	1	1	0	1	1	1	1	1	1	1	1	22	88%

(Wong & Figueroa, 2019) [16]	1	1	1	1	0	0	1	1	1	1	1	1	0	0	1	1	1	1	1	1	1	1	1	1	1	21	84%
(Rodrigues et al., 2020) [17]	1	1	1	1	1	1	1	0	1	1	1	1	1	1	1	1	1	1	1	0	1	0	1	1	1	22	88%
(Turri-Silva et al., 2020) [18]	0	1	1	1	1	0	1	1	1	1	1	1	1	1	0	1	1	1	1	1	1	1	1	1	1	22	88%
(Su et al., 2024) [19]	1	1	1	1	1	1	1	0	1	0	1	0	0	1	1	0	0	0	1	0	1	1	1	1	1	17	68%

Table S2_continue. Standard for Reporting Diagnostic Accuracy Studies Guidelines for Heart Rate Variability Research (STARD-HRV) [20].

SECTION AND TOPIC	No	ITEM
TITLE OR ABSTRACT	1	Identification as a study of validation.
ABSTRACT	2	Structured summary of study objective, design, methods, results, and conclusions.
INTRODUCTION	3	Scientific and practical background, including the intended use of the index device/software
	4	Study objectives and hypotheses described.
METHODS		
Study design	5	Study uses within-subject design.
	6	Intended sample size and how it was determined (e.g. G*Power 3)
Participants	7	Eligibility criteria including specific restrictions (medical use, gender, age, activity level or BMI).
Pre-Testing	8	Pre-testing guidelines reported (e.g., limitations to caffeine, alcohol, physical activity etc.).
Test methods	9	Setup of reference standard and index device described in sufficient detail to allow replication (e.g. hardware/software such as brand, electrode configuration, etc.).
	10	Description of environmental conditions (e.g. temperature, humidity, lights on or off, time of day) and posture.
	11	A stabilization period prior to sampling was described.
Interbeat interval	12	The raw sampling rate and length of collection are described.
	13	Acknowledgment of breathing (e.g. controlled or not controlled).
Analysis	14	Description of how estimates or comparison measures were calculated (e.g. ES, LOA, Pearson's r or ICC).
	15	Reasons for missing data, along with percentage missing (e.g., equipment, persistent ectopy) and how it was handled.
	16	Interbeat artifact identification method (e.g. algorithm, manual inspection).
	17	Artifact cleaning methods and percentage of beats corrected.
	18	Description of metrics used and software/script for HRV calculation (log transformation etc.).
	19	Specification of frequency bands used and how they were calculated (e.g. Fast Fourier Transform or Autoregressive modelling).
RESULTS		
Participants	20	Baseline demographics of participants.
Test results	21	Mean ± SD along with at least one estimates of precision (e.g. LOA, Pearson's r or ICC).
DISCUSSION		
	22	Study limitations, (e.g., sources of potential bias, confounding variables, statistical uncertainty, and generalizability).
	23	Implications for practice, including the intended use.

OTHER INFORMATION		
	24	Where the full study protocol can be accessed if not fully described.
	25	Sources of funding and other support; role of funders.

Table S3. Summary of the samples' characteristics included in the review.

**Reference**	***n***	**Sex**				**Mean age (years)**
		**Women**		**Men**		
(Gutin et al., 2000) [4]	79	53	67%	26	33%	9
(Facchini et al., 2003) [5]	40	30	75%	10	25%	30
(Stuckey et al., 2013) [6]	12	3	25%	9	75%	56
(Tian et al., 2015) [7]	82	33	40%	49	60%	42
(Farinatti et al., 2016) [8]	44	24	55%	20	45%	13 - 17
(Boudet et al., 2017) [9]	80	48	60%	32	40%	59
(Ramos et al., 2017) [10]	56	25	45%	31	55%	56
(Goit et al., 2018) [11]	41	41	100%	0	0%	44
(Kim et al., 2018) [12]	20	20	100%	0	0%	66
(Phoemsapthawee et al., 2019) [13]	21	0	0%	21	100%	20
(Vanzella, Dagostinho et al., 2019) [14]	52	NS				51
(Vanzella, Linares et al., 2019) [15]	53	NS				51
(Wong y Figueroa, 2019) [16]	20	20	100%	0	0%	54
(Rodrigues et al., 2020) [17]	70	47	67%	23	33%	43
(Turri-Silva et al., 2020) [18]	38	15	39%	23	61%	51
(Su et al., 2024) [19]	44	0	0%	44	100%	14
**Total**	**752**	**359**	**48%**	**288**	**38%**	**43**

NS: not specified (105 — 14% of the total sample)

References

1. Malik M et al. 1996 Heart rate variability: Standards of measurement, physiological interpretation, and clinical use. Circulation 93, 1043–65. https://doi.org/10.1161/01.cir.93.5.1043.
2. Shaffer F, Ginsberg JP. 2017 An Overview of Heart Rate Variability Metrics and Norms. Front Public Health. 5. https://doi.org/10.3389/fpubh.2017.00258.
3. Tarvainen MP, Niskanen JP, Lipponen JA, Ranta-Aho PO, Karjalainen PA. 2008 Kubios HRV - A Software for Advanced Heart Rate Variability Analysis. In IFMBE Proceedings. https://doi.org/10.1007/978-3-540-89208-3_243.
4. Gutin B, Barbeau P, Litaker MS, Ferguson M, Owens S. 2000 Heart rate variability in obese children: Relations to total body and visceral adiposity, and changes with physical training and detraining. Obes Res 8. https://doi.org/10.1038/oby.2000.3.
5. Facchini M, Malfatto G, Sala L, Silvestri G, Fontana P, Lafortuna C, Sartorio A. 2003 Changes of autonomic cardiac profile after a 3-week integrated body weight reduction program in severely obese patients. J Endocrinol Invest 26. https://doi.org/10.1007/BF03345142.
6. Stuckey MI, Kiviniemi AM, Petrella RJ. 2013 Diabetes and technology for increased activity study: the effects of exercise and technology on heart rate variability and metabolic syndrome risk factors. Front Endocrinol (Lausanne) 4, 121. https://doi.org/10.3389/fendo.2013.00121.
7. Tian Y, Huang C, He Z, Hong P, Zhao J. 2015 Autonomic function responses to training: Correlation with body composition changes. Physiol Behav 151. https://doi.org/10.1016/j.physbeh.2015.07.038.
8. Farinatti P, Neto SRM, Dias I, Cunha FA, Bouskela E, Kraemer-Aguiar LG. 2016 Short-term resistance training attenuates cardiac autonomic dysfunction in obese adolescents. Pediatr Exerc Sci 28. https://doi.org/10.1123/pes.2015-0191.
9. Boudet G et al. 2017 Paradoxical dissociation between heart rate and heart rate variability following different modalities of exercise in individuals with metabolic syndrome: The RESOLVE study. Eur J Prev Cardiol 24, 281–296. https://doi.org/10.1177/2047487316679523.
10. Ramos JS et al. 2017 High-intensity interval training and cardiac autonomic control in individuals with metabolic syndrome: A randomised trial. Int J Cardiol 245, 245–252. https://doi.org/10.1016/j.ijcard.2017.07.063.
11. Goit RK, Pant BN, Shrewastwa MK. 2018 Moderate intensity exercise improves heart rate variability in obese adults with type 2 diabetes. Indian Heart J 70, 486–491. https://doi.org/10.1016/j.ihj.2017.10.003.
12. Kim J, Park HY, Lim K. 2018 Effects of 12 weeks of combined exercise on heart rate variability and dynamic pulmonary function in obese and elderly Korean women. Iran J Public Health 47.
13. Phoemsapthawee J, Prasertsri P, Leelayuwat N. 2019 Heart rate variability responses to a combined exercise training program: Correlation with adiposity and cardiorespiratory fitness changes in obese young men. J Exerc Rehabil 15. https://doi.org/10.12965/jer.1836486.243.
14. Vanzella LM et al. 2019 Periodized Aerobic Interval Training Modifies Geometric Indices of Heart Rate Variability in Metabolic Syndrome. MEDICINA-LITHUANIA 55. https://doi.org/10.3390/medicina55090532.
15. Vanzella LM et al. 2019 Effects of a new approach of aerobic interval training on cardiac autonomic modulation and cardiovascular parameters of metabolic syndrome subjects. Arch Endocrinol Metab 63, 148–156. https://doi.org/10.20945/2359-3997000000111.
16. Wong A, Figueroa A. 2019 The effects of low-intensity resistance exercise on cardiac autonomic function and muscle strength in obese postmenopausal women. J Aging Phys Act 27. https://doi.org/10.1123/japa.2018-0418.
17. Rodrigues JAL, Ferrari GD, Trapé ÁA, de Moraes VN, Gonçalves TCP, Tavares SS, Tjønna AE, de Souza HCD, Júnior CRB. 2020 β2 adrenergic interaction and cardiac autonomic function: effects of aerobic training in overweight/obese individuals. Eur J Appl Physiol 120. https://doi.org/10.1007/s00421-020-04301-z.
18. Turri-Silva N et al. 2020 Functional Resistance Training Superiority Over Conventional Training in Metabolic Syndrome: A Randomized Clinical Trial. Res Q Exerc Sport 91, 415–424. https://doi.org/10.1080/02701367.2019.1679333.

19. Su Z, Yu W, Yan Z, Ding D, Fang C, Luo Q, Liu X, Cao L. 2024 Comparison of high-intensity interval training and moderate-intensity continuous training on cardiopulmonary function, cardiac autonomic function and vascular function in adolescent boys with obesity: A randomized controlled trial. Eur J Sport Sci 24, 1871–1882. https://doi.org/10.1002/ejsc.12207.
20. Dobbs WC, Fedewa M V., MacDonald H V., Holmes CJ, Cicone ZS, Plews DJ, Esco MR. 2019 The Accuracy of Acquiring Heart Rate Variability from Portable Devices: A Systematic Review and Meta-Analysis. Sports Medicine. 49. https://doi.org/10.1007/s40279-019-01061-5.
